# On the Formation of False Membranes within the Bladder from Cantharides Blisters Applied to the Skin

**Published:** 1844-12-28

**Authors:** 


### ON THE FORMATION OF FALSE MEMBRANES WITHIN THE BLADDER FROM CANTHARIDES BLISTERS APPLIED TO THE SKIN.

BY DR. MOREL-LAVELLEE.

Dr. Morel-Lavellee has recorded four cases of the formation of false membranes within the bladder in consequence of blisters of cantharides applied to the skin. In some individuals the tendency of the inflammatory affection of the bladder and the formation of false membranes was so great, that in the second case he records the smallest flying blister of cantharides, applied to the forehead, excited the inflammatory affection as often as it was applied. The symptoms attending the complaint are, frequent desire to micturate, pain at the *meatus urinarius* immediately after passing the last drops of water, the pain at length increasing so much that, when urine is passed, it gives rise to a burning sensation, as if the urine were melted lead. At length small soft pseudo-membranous pellets are expelled along with the urine. In the milder forms there is no fever, but in the more severe forms the fever is often intense, and the membranous substances obstruct the urethra, and are passed in long rolls; the urine is also highly albuminous. Copious emollient drinks, emollient cataplasms to the hypogastric region, or a hip-bath, and removal of the blister, in general, suffice to cure this affection of the bladder.—*Ibid, from L'Experience.*

